# Supplementation of a Homeopathic Complex in the Diet of Castrated Male and Female Nursery Piglets and Its Effects on Behavior

**DOI:** 10.3390/ani15131877

**Published:** 2025-06-25

**Authors:** Gustavo Zigovski, Isabela Cristina Colaço Bez, Mariana Regina Rosa Catoia, Amanda Gabriela Bickel, Ruan R. Daros, Kelly Mazutti Monteiro, Silvana Teixeira Carvalho, Paulo Levi de Oliveira Carvalho, Leandro Batista Costa

**Affiliations:** 1 Graduate Program in Animal Science, School of Medicine and Life Sciences, Pontifícia Universidade Católica do Paraná, Imaculada Conceição Street, 1155-Prado Velho, Curitiba 80215-901, PR, Brazil; gustavozipaula@gmail.com (G.Z.); isabela.bez@pucpr.edu.br (I.C.C.B.); m.catoia@hotmail.com (M.R.R.C.); r.daros@pucpr.br (R.R.D.); kelly.mazutti@pucpr.br (K.M.M.); 2 Monohub—Research Group for Monogastric Animals, Pontifícia Universidade Católica do Paraná, Curitiba 80215-901, PR, Brazil; 3 EthoLab—Applied Ethology and Animal Welfare Lab, Pontifícia Universidade Católica do Paraná, Curitiba 80215-901, PR, Brazil; 4 Animal Science Department, Universidade Estadual do Oeste do Paraná, Pernambuco Street, 1777-Centro, Marechal Cândido Rondon 85960-000, PR, Brazil; agbickel22@gmail.com (A.G.B.); silvana.carvalho@unioeste.br (S.T.C.); paulo.carvalho@unioeste.br (P.L.d.O.C.)

**Keywords:** behavioral tests, exploration, fear, homeopathy, null results, pigs, sex, stress

## Abstract

Homeopathy is widely used as a complementary therapy, but its effects on the behavior of production animals remain underexplored. This study evaluated the influence of a homeopathic complex on the behavior of castrated male and female piglets in the nursery phase. The experiment lasted 35 days and involved 105 animals. Piglets were allotted to five treatments in a completely randomized block design with seven replicates using three piglets per experimental unit. Tested treatments were: negative control—basal diet without additives; basal diets with 4.5, 6.0, 7.5, and 9.0 kg/ton of the homeopathic complex in the feed. Behavioral tests included open field (OF), novel object (NO), sociability, discriminative learning, judgment bias, and reactivity during weighing (RDW). Vocalizations were lower in females than males during the OF test (*p* = 0.016). In the RDW test, a trend was identified (*p* = 0.076): as the level of the homeopathic complex increased, escape attempts decreased in females and increased in males (sex × treatment interaction). Females also showed greater resistance to movement (*p* = 0.018). Our study suggests that the homeopathic complex does not impact the behavior of castrated male and female nursery piglets. The findings further illustrate that the sex of the animals affects their behavior, with females displaying higher levels of inactivity compared to castrated male piglets.

## Simple Summary

Homeopathy is often used alongside conventional treatments in both humans and animals. However, little is known about how it affects the behavior of farm animals. This study investigated whether adding a homeopathic complex to the feed of young piglets would change their behavior. To evaluate this, 105 piglets were divided into five treatment groups with increasing levels of the homeopathic complex, ranging from 0 to 9.0 kg per ton of feed. These animals underwent behavioral tests, and it was hypothesized that piglets receiving the homeopathic complex would show less fear and anxiety during the tests due to the physiological effects associated with the homeopathic compounds. At the end of the experiment, no significant effect of the homeopathic complex on piglet behavior was found. However, it was observed that the sex of the piglets plays an important role in how they respond to challenges, with females showing more inactivity than males. This knowledge can help farmers and researchers better understand and care for animals in production systems.

## 1. Introduction

Stress is a biological response experienced by piglets during the weaning period. Events such as separation from the sows, along with changes in the environment or diet, constitute challenging scenarios capable of inducing behavioral alterations [1]. Such stressors can also reduce animal productivity [2] and promote fear and anxiety [3]. Therefore, identifying compounds that reduce piglet stress and fear while promoting exploratory behavior is crucial for improving their welfare. In the search for a solution to address this demand, we identified homeopathy as a potential approach.

Homeopathy is a therapy that uses the principle of similarity: compounds capable of inducing symptoms (in high doses) have the potential to treat a disease with comparable symptoms (when administered in small doses) [4]. An advantage of homeopathic remedies is that, being ultra-diluted mixtures of animal, vegetable, and mineral components, they do not leave contaminating residues in animal-derived products or in the environment [5], making them a potential alternative for use as animal additives and behavior modulators.

The use of homeopathy in piglets has been studied, but mainly to verify its effects on performance [6], prevention of diarrhea [7], and gut health [8]. Currently, there are few experiments evaluating its effects on the behavior of castrated male and female piglets, although exploring this field is necessary due to the behavioral and psychological effects associated with homeopathic treatments observed in other animal species [9,10,11]. *Chamomilla*, for example, has already been associated with sedative and anxiolytic effects in calves [12]. Another compound, *Staphysagria*, has been linked to antidepressant properties, particularly in rodents [13]. Moreover, studies conducted in mice have demonstrated the anxiolytic and emotional response–modulating effects of *Ignatia amara* and other homeopathic remedies [10,14]. This entire context, mostly involving the sole use of laboratory animal models, led us to investigate the possible effects that a complex containing these and other homeopathic medicines could have on the behavior of piglets in the nursery phase, especially as this is a very stressful period for these animals [1]. The combined use of several of these components may positively influence the piglets’ behavioral state. Additionally, identifying potential differences in effects between castrated males and females is also important since the sex of the animals has already been shown in the literature to influence their behavioral tendencies [8,15,16,17]. Therefore, in the present study, the behavioral comparison between castrated males and females was also evaluated. The outcomes may complement the literature by providing additional data for future experiments.

Considering these aspects, the aim of the present research was to evaluate the effect of a homeopathic complex on the behavior of castrated male and female nursery piglets. We hypothesized that, due to the anxiolytic, sedative, and emotional response–modulating effects associated with homeopathic treatments reported in the literature, the homeopathic complex would influence animal behavior, leading piglets to exhibit fewer behaviors associated with fear and anxiety while also potentially enhancing their learning abilities and cognitive bias.

## 2. Materials and Methods

### 2.1. Animals, Experimental Design, and Housing

This study was approved by the Ethics Committee on Animal Use (CEUA) of the Pontifical Catholic University of Parana under protocol number 01720—2nd version. Animals were handled only when necessary and exclusively by trained and qualified personnel. The piglets were not subjected to any unnecessary discomfort throughout the experimental period. There were no animal losses throughout the entire experiment.

This research is part of a broader project encompassing more than just animal behavior. Consequently, a power calculation was not performed for the objectives of the current study. Nevertheless, the literature reports statistical differences observed in studies with sample sizes comparable to those employed in the present experiment [18,19]. Furthermore, in the behavioral study by Clouard and colleagues (2022) [20], a minimum sample size of five animals in each treatment would already have sufficient power to detect differences between groups.

In the present randomized trial, a total of 105 crossbred piglets (31.5% Pietrain × 25% Landrace × 34.25% Large White × 9.25% Duroc), consisting of 54 castrated males and 51 females, weaned at 21 ± 1.3 d, with an initial weight of 5.56 ± 1.15 kg, were assigned to five treatments in a completely randomized block design with seven repetitions and three piglets per experimental unit (distributed according to their weight and sex). The experiment lasted 35 days. The piglets were housed at the Piglet Research Unit of the Pontifical Catholic University of Paraná, located in Fazenda Rio Grande (Paraná, Brazil) in suspended pens with metal feeders, water dispensers, and plastic slatted floors. From the time the animals arrived at the facility until the start of the study with the experimental diets (4 days), all piglets received a basal diet. From the 5th day on, each animal group received its specific treatment. The treatments were composed of five experimental diets: negative control—basal diet without additives (NC), and basal diets with 4.5, 6.0, 7.5, and 9.0 kg/ton of a homeopathic complex in the feed. Feed and water were provided ad libitum. The treatments received by the animals were stored in bags placed inside fiber drums positioned in front of the pens, which were opened only at feeding time. Only the research supervisor had knowledge of the treatments and was responsible for their random allocation in the fiber drums. All other researchers involved in animal management, including the statistician, were blinded to the treatment assignments. Treatments were revealed only after data analysis to allow for the discussion of the present study.

### 2.2. Composition of the Homeopathic Complex

The homeopathic complex was composed of: Ignatia amara (30 cH), Chamomilla (15 cH), Silicea terra (15 cH), Staphysagria (15 cH), Colibacillinum (9 cH), Enterococcinum (9 cH), Lac defloratum (9 cH), Aethusa cynapium (6 cH), Artemisia abrotanum (6 cH), Calcarea carbonica (6 cH), Thyreoidinum (6 cH), Thymulin (6 cH), and Vehicle (Calcium Carbonate—1000 g). Most of these remedies are indicated as anxiolytics, antidepressants, and immunomodulators. The complex was provided by Real H Company—Animal Nutrition and Health (Campo Grande, Mato Grosso do Sul, Brazil). Its use is intended for piglets, and there is no contraindication. The diets were formulated (Supplementary Table S1) to meet the nutritional requirements of the piglets: pre-starter I (1 to 7 d of experimentation), pre-starter II (8 to 20 d of experimentation), and starter (21 to 37 d of experimentation) phases, following Rostagno and colleagues (2017) [21].

### 2.3. Pre-Dietary Treatments Observations

Immediately after the distribution of the piglets into their respective pens, while receiving the basal diet, agonistic interactions were observed for 2 min (for each pen). They were categorized as negative interactions and fights between animals. The author, the receptor (target), and the winner of the agonistic interaction were recorded. The observations were performed at a safe distance that did not compromise the animals’ behavior due to human presence and were done by the same two researchers with prior training. A total of 10 rounds of observations were conducted over 3 days, with each pen observed for a total of 20 min. At the end of the 1st and 2nd days, the number of skin lesions of each animal was recorded. These procedures were performed to identify the dominant and subordinate piglets in each pen. The imposition of dominance is done through aggressive behaviors, and defensive behaviors are performed by subordinate piglets [22]. By observing agonistic behaviors, the number of fights each animal initiated and won, as well as the presence of skin lesions, it was possible to presume which piglet was dominant in each pen. Animals that were the target of fights were considered subordinates. In order to avoid the influence of piglets’ hierarchical status in certain behavioral tests, we chose to standardize the hierarchical group by using only the dominant animals from each pen.

### 2.4. Behavioral Tests

**Figure 1:**
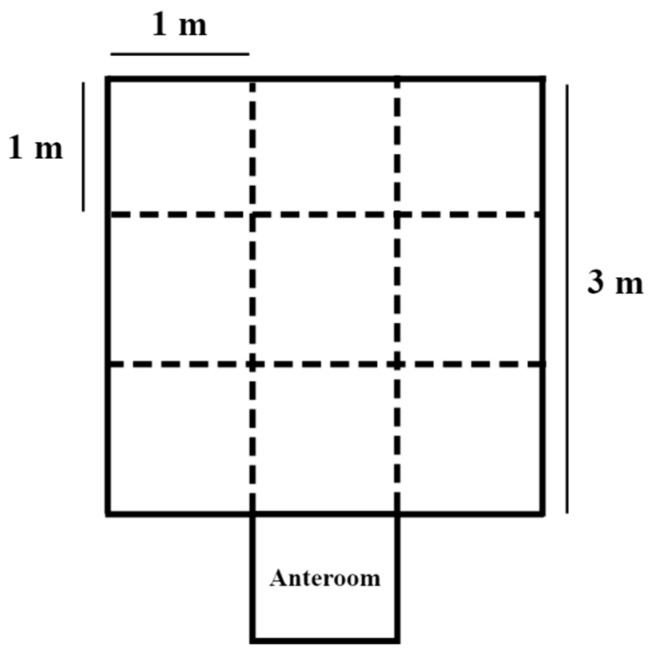
Arena used for open field, novel object, sociability, discriminative learning, and judgment bias behavioral tests in piglets that received or did not receive a homeopathic complex in the diet.

Behavioral tests were applied when the piglets reached 37 days of age (d 16 of experiment). All tests, except reactivity during weighing, were carried out in a 9.00 m^2^ arena, with nine quadrants of 1 m^2^ each marked out on the ground (Figure 1). The animals were transported to the arena using a metal cart, with minimal contact with the researchers. The description of the behaviors assessed during the behavioral tests conducted after the provision of treatments to the piglets can be seen in Table 1.

### 2.5. Open Field and Novel Object Tests

On d 16, in the morning (0900 to 1030 h), the dominant piglet of each pen (total n = 35) was taken to a structure that served as an anteroom of the unknown environment (arena) for the open-field test (OF). After releasing the animal into the arena, different behaviors were observed for 2 min: quantity of quadrants explored, time spent exploring the arena, and vocalizations. The piglet was then returned to its pen. On the same day as the OF test, in the afternoon (1400 to 1530 h), the novel object test (NO) was performed with the same animals (total n = 35). For this test, an object, that had never been seen by the piglets (a conventional blue cement feeder), was placed in the center of the arena. For 2 min after the animals were released from the anteroom, the researchers recorded the frequency and duration of the piglets’ contact with the object. The procedures and parameters used for these two tests were adapted from Puppe and colleagues (2007) [23] and Haigh and colleagues (2020) [24].

### 2.6. Sociability Test

In this test, the objective was to evaluate the aggressive behavior that two dominant piglets from the same treatment, but from different pens, would display when placed in the behavioral arena. The environment was already familiar to them due to previous tests, and to better understand the animals’ reactions, chopped apples were placed in the center of the arena to serve as a potential resource for competition. The test was performed on d 17 of the experiment (0900 to 1030 h), and it was conducted with pairs of dominant piglets (n = 30). The behavioral test lasted 3 min, and the researchers observed fighting, escape attempts, negative interactions, and eliminatory behaviors.

### 2.7. Discriminative Learning

**Figure 2:**
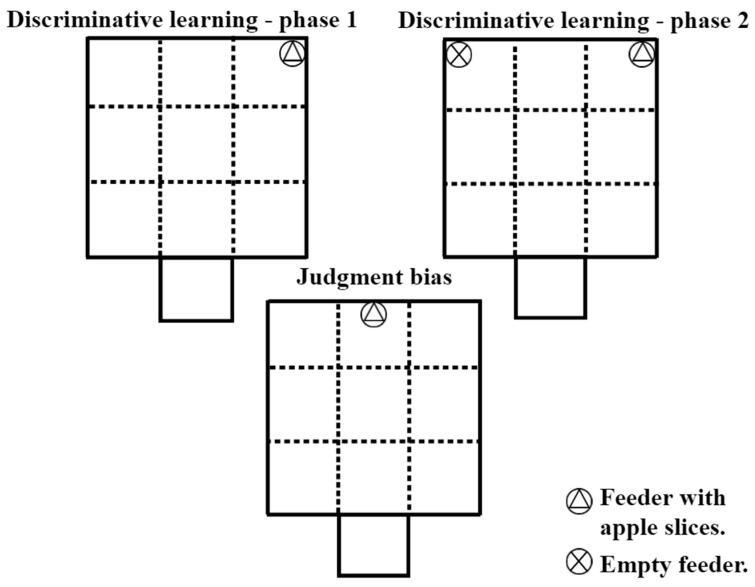
Positioning of the feeders for the discriminative learning and judgment bias tests.

From d 23 onwards, discriminative learning test was performed in two phases and both lasted 2 min for each session/piglet. For this test, the 35 dominant animals were used, and the sessions were held daily in the mornings and afternoons (0900 to 1200 h and 1400 to 1700 h). During phase one, a feeder (containing apple slices) was put on one side of the arena and the opposite side remained empty. When the piglet was released in the arena, the type of interaction with the feeder/apple (did not touch the feeder; touched the feeder; ate the apple) and number of attempts/sessions to reach the feeder were observed (each instance in which the piglet was placed in the arena was considered a single attempt). For the piglet to move on to the next phase, it had to meet the criterion of three consecutive sessions eating the apple or touching the feeder. During phase two, two feeders were placed in the arena on opposite sides, with one of them containing apple slices (positive reinforcement), and the other had no content (positive punishment). If the animal encountered the empty feeder, an aversive crumpling sound was performed as punishment. The following variables were recorded: type of interaction with the feeder, number of attempts/sessions, and number of punishments. For the piglet to proceed to the judgment bias test, it had to meet the criterion of three consecutive sessions eating the apple or touching the correct feeder without receiving punishment. A more detailed representation of the feeder positions for these tests is shown in Figure 2.

### 2.8. Judgment Bias Test

The judgment bias test was performed on d 35 of the experiment in the morning (0900 to 1000 h) and lasted 1 min per piglet. Each animal underwent a single session. During the test, only one feeder (containing apple slices) was placed in an ambiguous location, positioned between the previously learned zones of positive reinforcement and positive punishment (Figure 2). Researchers observed the piglets’ interaction with the feeder, categorizing the behavior into one of three responses: no contact with the feeder, touching the feeder, or consuming the apple. Both touching the feeder and consuming the apple were considered optimistic (positive) responses, whereas not approaching the feeder was interpreted as a pessimistic (negative) response.

### 2.9. Reactivity During Weighing

The piglets’ reactivity was evaluated while they were being weighed during the animals’ feed change on d 7, 21, and 35 of the experiment. On these days, the animals were placed in a large plastic box that was on a scale so that the observers could assess the animals’ behavior for 1 min (n = 105). During the observation period, researchers maintained an average distance from the piglet to assess the behaviors without influencing the measured parameters due to human presence. The evaluated behaviors included: vocalizations, escape attempts, and resistance to movement [25,26,27].

### 2.10. Statistical Analysis

For all outcomes of interest, homeopathic levels and sex were included as factors, as well as their interaction. The blocking factor was used solely for animal allocation and, thus, was not included in the statistical model. When repeated measures were used, animal identification was included as a random effect in the generalized linear models (GLMs). Model assumptions were verified via residual diagnostic plots, including residuals versus fitted values to assess homoscedasticity and Q-Q plots to check the normality of residuals. Regarding the behaviors in the OF and NO tests, linear regression was performed. A mixed linear regression was performed for the reactivity test during weighing, as well as for sociability and discriminative learning. *p* values less than 0.05 were considered significant, while 0.05 ≤ *p* < 0.10 indicated a trend in the data. R Core Team statistical software (version 4.5.0) was used for all behavioral tests [28].

## 3. Results

No piglets were removed or excluded from the study for any reason. The results of all the behavioral tests can be seen in Table 2.

### 3.1. Open-Field Test

Vocalizations were affected by sex, with castrated male piglets vocalizing more than females regardless of the treatment provided (*p* = 0.016). The mean number of vocalizations for castrated males was 40.4 (±5.69), and for females, was 19.4 (±5.86). Explored quadrants and time exploring the arena showed no statistical differences (*p* > 0.05).

### 3.2. Novel Object Test

No statistical differences were observed for any of the analyzed variables for treatments or sex (*p* > 0.05).

### 3.3. Sociability

No effect of treatments or sex was found for negative interactions (*p* > 0.05). We did not have sufficient data to perform statistical tests for escape attempts, fights, and eliminatory behaviors; these parameters were not consistently observed.

### 3.4. Discriminative Learning and Judgment Bias Test

There was no statistical difference between treatments or sex in the number of attempts during phases 1 and 2, or in the number of punishments in the discriminative learning test (*p* > 0.05). For the judgment bias test, of the 35 animals, 12 reached the learning criteria, passing to the judgment bias, presenting, for these piglets, an average of 10.6 (±0.60) attempts to reach this threshold. For the judgment bias test, we did not have enough data that allowed statistical analysis, since a minimal number of animals reached this phase. However, descriptively, it was observed that only two animals touched the feeder (treatments NC and 9.0), while the others did not exhibit this behavior.

### 3.5. Reactivity During Weighing

**Figure 3:**
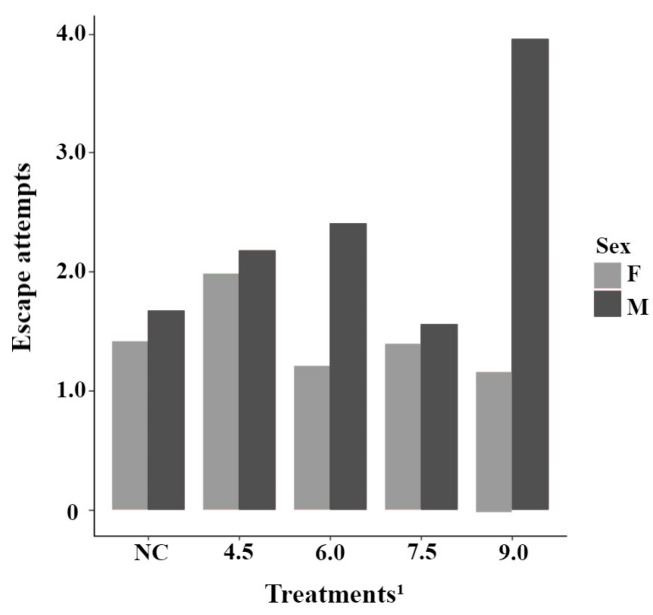
Interaction between homeopathic complex in the diet and sex for escape attempts for reactivity during weighing test performed on nursery piglets. ^1^ Negative control—basal diet without additives (NC), basal diets with 4.5; 6.0; 7.5 and 9.0 kg/ton of homeopathic complex in the feed. Sex: F—Female, M—Male.

There was a statistical trend for the interaction between treatment and sex for escape attempts (*p* = 0.076). As the quantity of the homeopathic complex in the diet increased, castrated male piglets tended to show a higher number of escape attempts, whereas females showed a lower number (Figure 3). A statistical difference in resistance to movement concerning sex (*p* = 0.018) was also observed. The mean resistance to movement was 50.4 (±0.59) for castrated males and 52.4 (±0.60) for females. Vocalizations showed no statistical differences (*p* > 0.05).

## 4. Discussion

The results obtained in this study showed that the levels of the homeopathic complex did not influence the behaviors evaluated in the different behavioral tests, at least when considered separately. Nevertheless, adding the homeopathic complex in greater quantities to the diets seems to be associated with the sex of the animals; higher levels led to a trend of females showing fewer escape attempts. The effect was the opposite for males, as their escape attempts increased. This trend observed in escape attempts, at least in females, could be explained by the compounds present in the complex provided to the animals. *Chamomilla*, for example, has stress-modulating properties, accelerating the recovery of basal behavior in animals subjected to stressful situations [9]. Its use can bring additional benefits, as it has sedative, analgesic, and anxiolytic effects [12]. Despite the positive effects mentioned for females, the increase in escape attempts observed in males may be directly related to the greater propensity of this sex to be more active, showing more involvement in social play episodes and a higher number of agonistic interactions compared to females [15]. Clouard and colleagues (2022) [15], when observing behavioral differences between male and female piglets, suggested that these discrepancies are associated with the sexual dimorphism of the animals, which influences how each sex expresses its social behaviors. It is important to emphasize that, in many of these studies, the males were intact, and it is known that castration can potentially alter certain behaviors in swine [29,30]. Nevertheless, in the present study, castrated males exhibited the same behavioral patterns as intact males reported in the literature.

The other outcomes found in this research clearly demonstrated a behavioral difference associated with the sex of the animals. Interestingly, our study is not the first to suggest a sex-related difference in the behavior of piglets. The literature indicates that factors such as age and sex may influence behavioral responses [17]. One of the explanations found for these behavioral discrepancies between males and females is the different brain development between the sexes. Researchers reached this conclusion by observing how the piglets exhibited different behaviors in response to a new stimulus [16]. Female piglets, in a study that intended to explore social behaviors, were considered more inactive when compared to males, since males were responsible for greater behavioral expressions, such as agonistic and social interactions [15]. The resistance to movement evaluated in the present research highlights this characteristic of females to be more inactive.

Other homeopathic remedies in the complex, such as *Calcarea carbonica* and *Staphysagria*, are known for their anxiolytic and antidepressant properties [13,31]. These remedies led us to believe that we would find differences in the behavior of animals. An investigation conducted on rodents to assess the effects of *Staphysagria* highlighted its considerable antidepressant attributes [13]. Some flavonoids present in this compound have been associated in previous studies with increased serotonin levels, which may contribute to the modulation of mood-related behaviors [13,32]. However, it is important to note that such mechanisms were not directly evaluated in the present study.

Regarding the OF test, females showed fewer vocalizations than males, which provides further evidence of the sex effect in behavioral tests [17]. For this parameter, the homeopathic complex had no influence. The variables directly related to exploration were also not affected by any level of the homeopathic complex. The absence of effect on this set of behaviors has already been reported in the literature. In order to verify some emotion-related symptoms in mice, *Ignatia amara* has been used intraperitoneally in this species, and the results were compared with an allopathic drug [10]. The authors noted that the time spent by the mice in the center of the arena was not influenced by either the homeopathic remedy or the allopathic remedies.

Concerning the sociability test, it was expected that the piglets receiving homeopathic complex levels could display fewer aggressive behaviors, escape attempts, and eliminatory behaviors compared to the animals in the negative control. This hypothesis was due to the significant presence of anxiolytic remedies within the homeopathic complex. *Aethusa cynapium* serves as an example, with its mechanism of action associated with fatty acids while also playing a role in anxiety regulation [33]. Once the animals from the negative control group did not exhibit significant levels of aggressive behavior, the minimal observation of fights in the present study may be explained by the animals’ observation period. To circumvent this issue, the feeder placed at the center of the arena with sliced apples would serve as a stimulus for animal interaction. In piglets, hierarchies typically form within 3 days [34], requiring a substantial observation period to witness behaviors related to escape attempts or negative interactions. For future experiments, it is recommended that piglets undergo a prior fasting period before the test. This approach will encourage competition for food. Furthermore, a longer testing period could be considered in future studies to determine whether behaviors that were rarely observed (such as escape attempts, fights, and eliminatory behaviors) might occur more frequently under extended observation.

In relation to discriminative learning, the literature indicates that with at least 10 sessions/attempts, most pigs can learn to discriminate between rewarded and unrewarded cues, mainly based on location [35]. In our study, the average number of sessions was lower. However, this limited number of sessions did not prevent 12 animals from moving on to the next stage, which was the judgment bias test. The homeopathic complex and the piglets’ sex did not appear to have an impact on these last two tests conducted in the arena. It is important to emphasize that, for the judgment bias test, it has already been described that sex did not influence the animals [36,37]. Regarding these tests, we acknowledge the low number of animals used as a methodological limitation. Considering that these are more complex tests that require longer time to complete, a larger number of piglets could increase the robustness of the results and the precision of the inferences drawn.

More studies involving homeopathic complexes should be conducted, especially including a larger sample size and all hierarchical groups. In addition, different levels or combinations of homeopathic potencies could be tested, since it is known that the potency used and experimental conditions can influence the effects of the tested remedies on the animals [38].

## 5. Conclusions

The present study did not show results that demonstrate the effect of the homeopathic complex on the behavior of the animals. The sex of the piglet was found to influence the parameters of vocalizations in the open-field test and resistance to movement in the reactivity during weighing test. These findings suggest that female piglets exhibited a less active behavioral profile than castrated males under the studied conditions. Further studies are recommended to assess different homeopathic levels and behavioral endpoints across broader piglet populations.

## Figures and Tables

**Table 1:** Ethogram used to evaluate piglets in the nursery phase with or without a homeopathic complex in their diet.

Behavior	Description	Tests
Explored quadrants	Number of quadrants explored (quantity)	Open Field
Time exploring the arena	Time spent exploring the arena (seconds)	Open Field
Vocalizations	Screams, squeals, or grunt-squeals (quantity)	Open Field and Reactivity during weighing
Frequency	Number of times the piglet touched the object (quantity)	Novel Object
Duration of contact	Contact time with the object (seconds)	Novel Object
Fighting	Fight between two piglets (seconds)	Sociability
Negative interactions	Ear or tail biting (quantity)	Sociability
Eliminatory behaviors	Defecating or urinating (yes or no)	Sociability
Escape attempts	Number of times the animal jumped/raised its front legs against the sides of the weighing scale (quantity)	Sociability and Reactivity during weighing
Type of interaction with the feeder	Interaction or lack of interaction with the feeder/apple (did not touch the feeder; touched the feeder; ate the apple)	Discriminative learning (phase one and two) and Judgement bias
Number of punishments	Number of times the punishment was applied (quantity)	Discriminative learning (phase two)
Resistance to movement	No movement of any portion of the piglet’s body visible for a period > 2 s. The duration comprises the beginning of the standstill to any movement of the body (seconds)	Reactivity during weighing

**Table 2:** Mean values (±standard error) from behavioral tests performed on nursery piglets with or without a homeopathic complex in their diet.

Items	Treatments ^1^					*p*-Value		
	NC	4.5	6.0	7.5	9.0	T ^2^	S ^3^	T × S
Open Field (n = 35)								
Vocalizations (Quantity) *	41.1 (±9.1)	18.6 (±9.1)	25.4 (±9.1)	36.6 (±9.1)	27.6 (±9.1)	0.435	0.016	-
Explored quadrants (Quantity)	14.4 (±2.6)	17.3 (±2.6)	13.7 (±2.6)	19.7 (±2.6)	14.0 (±2.6)	0.429	0.349	-
Time exploring the arena (Seconds)	48.9 (±5.5)	59.6 (±5.5)	51.2 (±5.5)	61.2 (±5.5)	49.4 (±5.5)	0.358	0.364	-
Novel Object (n = 35)								
Frequency (Quantity)	1.6 (±0.6)	0.8 (±0.6)	1.1 (±0.6)	1.4 (±0.6)	1.4 (±0.6)	0.876	0.591	-
Duration of contact (Seconds)	7.5 (±4.3)	4.9 (±4.3)	6.0 (±4.3)	12.9 (±4.3)	10.1 (±4.3)	0.711	0.896	-
Reactivity During Weighing (n = 105)								
Escape attempts (Quantity)	0.9 (±0.2)	1.3 (±0.2)	1.2 (±0.2)	0.8 (±0.2)	1.1 (±0.2)	0.629	0.651	0.076
Vocalizations (Quantity)	10.1 (±2.0)	14.7 (±2.0)	17.0 (±2.1)	11.2 (±2.0)	13.3 (±2.0)	0.117	0.422	-
Resistance to movement (Seconds) *	53.3 (±0.9)	52.1 (±0.9)	50.4 (±0.9)	50.9 (±0.9)	50.1 (±0.9)	0.089	0.018	-
Sociability (n = 30)								
Negative interactions (Quantity)	2.1 (±1.4)	3.0 (±1.4)	1.1 (±1.4)	4.0 (±1.4)	1.5 (±1.4)	0.649	0.266	-
Discriminative learning behavior (n = 35)								
Number of attempts on phase 1 (Quantity)	2.6 (±0.3)	3.1 (±0.2)	2.7 (±0.2)	2.6 (±0.3)	2.8 (±0.2)	0.778	0.617	-
Number of attempts on phase 2 (Quantity)	7.8 (±0.3)	7.2 (±0.3)	7.4 (±0.3)	6.7 (±0.3)	6.8 (±0.3)	0.113	0.375	-
Number of punishments (Quantity)	0.5 (±0.1)	0.4 (±0.1)	0.4 (±0.1)	0.5 (±0.1)	0.4 (±0.1)	0.920	0.698	-

^1^ Negative control—basal diet without additives (NC), basal diets with 4.5, 6.0, 7.5, and 9.0 kg/ton of homeopathic complex in the diet. ^2^ The effects of treatments. ^3^ The effects of sex. * Significant statistical difference for sex.

## Data Availability

The data that support the findings of this study are available from the corresponding author upon reasonable request.

## Supplementary Materials

The following supporting information can be downloaded at: https://www.mdpi.com/article/10.3390/ani15131877/s1, Supplementary Table S1: Composition of the experimental diets fed to nursery piglets (as fed, g per kg).
