# Supplementary material for: Supplementation of a Homeopathic Complex in the Diet of Castrated Male and Female Nursery Piglets and Its Effects on Behavior
Source: Animals (Basel). 2025 Jun 25;15(13):1877. doi: 10.3390/ani15131877 (PMC12249404; doi:10.3390/ani15131877)
Supplement: Supplementary file 1 [file animals-15-01877-s001.zip › animals-3700784-supplementary.pdf]

Supplementary Table S1
Composition of the experimental diets fed to nursery piglets (as fed, g per kg).

Item	Treatments[1] - Pre starter I phase					Treatments[1] - Pre starter II phase					Treatments[1] - Starter phase				
	NC	4.5	6.0	7.5	9.0	NC	4.5	6.0	7.5	9.0	NC	4.5	6.0	7.5	9.0
Ingredients (%)															
Corn 8.00% CP	40.69	39.80	39.51	39.21	38.92	50.71	49.82	49.53	49.23	48.93	61.13	60.24	59.95	59.65	59.36
Soybean meal 45.00% CP	21.02	21.16	21.20	21.25	21.29	19.38	19.52	19.56	19.61	19.65	23.68	23.82	23.87	23.91	23.96
Skim milk powder	14.02	14.02	14.02	14.02	14.02	8.92	8.92	8.92	8.92	8.92	-	-	-	-	-
Micronized soybeans	10.00	10.00	10.00	10.00	10.00	8.00	8.00	8.00	8.00	8.00	6.00	6.00	6.00	6.00	6.00
Sugar	5.00	5.00	5.00	5.00	5.00	4.00	4.00	4.00	4.00	4.00	-	-	-	-	-
Fishmeal	3.00	3.00	3.00	3.00	3.00	3.00	3.00	3.00	3.00	3.00	3.00	3.00	3.00	3.00	3.00
Soybean oil	1.95	2.25	2.35	2.45	2.55	1.77	2.07	2.17	2.27	2.38	2.12	2.42	2.52	2.62	2.72
Dicalcium phosphate	1.48	1.48	1.48	1.48	1.48	1.43	1.43	1.43	1.43	1.43	1.53	1.53	1.53	1.53	1.53
Limestone	0.84	0.84	0.84	0.84	0.84	0.75	0.75	0.74	0.74	0.74	0.68	0.68	0.68	0.68	0.68
Lysine sulfate 54.6%	0.71	0.71	0.71	0.71	0.71	0.72	0.72	0.72	0.72	0.72	0.57	0.56	0.56	0.56	0.56
L-threonine 98.0%	0.43	0.43	0.43	0.43	0.43	0.39	0.39	0.39	0.39	0.39	0.28	0.28	0.28	0.28	0.28
DL-methionine 99.0%	0.36	0.36	0.36	0.36	0.36	0.31	0.31	0.31	0.31	0.31	0.23	0.23	0.23	0.23	0.24
L-tryptophan 99.0%	0.10	0.10	0.10	0.10	0.10	0.09	0.09	0.09	0.09	0.09	0.06	0.06	0.06	0.06	0.06
Premix[2]	0.30	0.30	0.30	0.30	0.30	0.30	0.30	0.30	0.30	0.30	0.30	0.30	0.30	0.30	0.30
Salt	0.11	0.11	0.11	0.11	0.11	0.23	0.23	0.23	0.23	0.23	0.42	0.42	0.42	0.42	0.42
Ultra-diluted complex	-	0.45	0.60	0.75	0.90	-	0.45	0.60	0.75	0.90	-	0.45	0.60	0.75	0.90
Analyzed composition															
Dry matter (%)	90.74	91.87	90.29	91.97	91.69	89.62	91.03	89.77	88.60	88.89	90.12	89.58	89.50	88.70	89.07
Ether extract (%)	6.90	7.23	6.89	6.94	7.11	6.82	6.76	7.93	6.96	6.89	6.71	6.61	6.11	7.11	6.77
Calculated composition															
Metabolizable energy (Mcal/kg)	3.40	3.40	3.40	3.40	3.40	3.38	3.38	3.38	3.38	3.38	3.35	3.35	3.35	3.35	3.35
Crude protein (%)	21.42	21.42	21.42	21.42	21.42	19.87	19.87	19.87	19.87	19.87	20.55	20.55	20.55	20.55	20.55
Total calcium (%)	1.07	1.07	1.07	1.07	1.07	0.97	0.97	0.97	0.97	0.97	0.91	0.91	0.91	0.91	0.91
Available phosphorus (%)	0.53	0.53	0.53	0.53	0.53	0.48	0.48	0.48	0.48	0.48	0.45	0.45	0.45	0.45	0.45
Total sodium (%)	0.22	0.22	0.22	0.22	0.22	0.22	0.22	0.22	0.22	0.22	0.21	0.21	0.21	0.21	0.21
Digestible lysine (%)	1.45	1.45	1.45	1.45	1.45	1.35	1.35	1.35	1.35	1.35	1.28	1.28	1.28	1.28	1.28
Digestible methionine + cystein (%)	0.81	0.81	0.81	0.81	0.81	0.75	0.75	0.75	0.75	0.75	0.73	0.73	0.73	0.73	0.73
Digestible threonine (%)	0.97	0.97	0.97	0.97	0.97	0.90	0.90	0.90	0.90	0.90	0.83	0.83	0.83	0.83	0.83
Digestible tryptophan (%)	0.28	0.28	0.28	0.28	0.28	0.26	0.26	0.26	0.26	0.26	0.24	0.24	0.24	0.24	0.24

[1] Negative control – basal diet without additives (NC), basal diets with 4.5, 6.0, 7.5, and 9.0 kg/ton of homeopathic complex in the feed. [2] Vitamin A (min.) 4.000.000UI/kg, vitamin D_3 (min.) 800.000 UI/kg, vitamin E (min) 10.000UI/kg, vitamin K_3 (min.) 1.600 mg/kg, vitamin B_1 (min.) 800 mg/kg, vitamin B_2 (min.) 2.000 mg/kg, vitamin B_6 (min.) 800 mg/kg, vitamin B_{12} (min.) 8.000 mcg/kg, niacin (min.) 16 g/kg, pantothenic acid (min.) 8.000 mg/kg, folic acid (min.) 240 mg/kg, biotin (min.) 80 mg/kg, manganese (min.) 30 g/kg, zinc (min.) 40 g/kg, iron (min.) 30 g/kg, copper (min.) 5.000 mg/kg, iodine (min.) 500 mg/kg, selenium (min.) 150 mg/kg.
